# Molecular prevalence of *Borrelia burgdorferi, Ehrlichia canis*, and *Coxiella burnetii* in dogs and associated ticks in Egypt: Emerging One Health challenging zoonoses

**DOI:** 10.14202/vetworld.2024.2586-2594

**Published:** 2024-11-22

**Authors:** Zeinab S. Ahmed, Nada Hesham, Taher M. Abdelhamid, Mahmoud E. Hashad, Hossam Mahmoud

**Affiliations:** 1 Department of Zoonoses, Faculty of Veterinary Medicine, Cairo University, Giza, Egypt; 2 Department of Microbiology, Faculty of Veterinary Medicine, Cairo University, Giza, Egypt; 3 Department of Medicine and Infectious Diseases, Faculty of Veterinary Medicine, Veterinary Hospital, Cairo University, Giza, Egypt

**Keywords:** borreliosis, coxillosis, ehrlichiosis, pets, *Rhipicephalus*

## Abstract

**Background and Aim:**

Tick-borne pathogens pose a significant problem in canines, other animals, and humans worldwide. This study aimed to estimate the prevalence of *Borrelia burgdorferi, Ehrlichia canis*, and *Coxiella burnetii* in dogs and associated ticks in Egypt.

**Materials and Methods:**

Blood samples from 110 tick-infested dogs and 550 whole ticks (divided into 110 pools) were collected and tested for the targeted pathogens using polymerase chain reaction (PCR).

**Results:**

Of the 110 dog blood samples, *B. burgdorferi* DNA was detected in three samples, *E. canis* in six samples, and *C. burnetii* in one kenneled dog. Among the 110 tick pools, *B. burgdorferi* was detected in four pools, *E. canis* in 12 pools, and *C. burnetii* in three pools from kenneled dogs. The overall prevalence of the three agents in dog and tick samples were 3.18%, 8.18%, and 1.81%, respectively. Simultaneous positive PCR reactions in both dogs and their associated tick pools were observed in four cases. *B. burgdorferi* and *E. canis* were simultaneously detected in two dogs and two tick pools, whereas *C. burnetii* was detected in one dog but not in any tick pools. The three agents were simultaneously detected in one dog, but none were found in the corresponding tick pools. A mixed infection of *C. burnetii* and *B. burgdorferi* was observed in one dog and one tick pool.

**Conclusion:**

Molecular diagnosis is the most reliable method for detecting *B. burgdorferi, E. canis*, and *C. burnetii* in dogs and associated ticks. *E. canis* showed the highest prevalence in both dog and tick samples followed by *B. burgdorferi* while *C. burnetti* showed the lowest prevalence. The potential transmission of these diseases from companion dogs to humans through ticks presents a significant challenge for the One Health concept.

## Introduction

Animal health is closely linked to public health; both are pillars of the One Health concept. Blood-sucking ticks act as vectors for zoonotic pathogens, transmitting serious agents among both animal and human hosts. Although animals can maintain tick cycles without illness, they can be clinically affected by human tick-borne pathogens (TBPs) [1]. Tick-borne diseases negatively impact the animal and public health sectors globally. In addition to consuming large amounts of host blood, ticks transmit dangerous pathogens. Except for tropical regions, ticks are found worldwide, including in Egypt [2]. *Rhipicephalus sanguineus* (the brown dog tick) is a common species found in tropical countries [3]. Tick-borne diseases cause economic losses in animal production and may result in chronic debilitating diseases in humans and companion animals [4]. Ticks often carry several bacterial pathogens, of which *Borrelia burgdorferi, Ehrlichia canis*, and *Coxiella burnetii* are the most severe species [5]. The increasing numbers of stray dogs and cats and the increase in pet ownership create natural incubating niches for tick-borne zoonotic infectious agents [6].

*Ehrlichia* is a Gram-negative bacterium belonging to the *Anaplasmataceae* family and order *Rickettsiales*. *Ehrlichia* species are intracellular pathogens that prefer peripheral white blood cells of various mammalian species, resulting in a globally recognized zoonotic disease. Ehrlichiosis is typically encountered in hot regions of the world where the intermediate vector tick host species, *R. sanguineus* (family *Ixodidae*), exists [7–9]. In dogs, ehrlichiosis presents with non-specific clinical symptoms [10, 11]. The disease may manifest in humans with mild symptoms who do not require medical care. However, life-threatening forms of human ehrlichiosis, such as meningoencephalitis, acute respiratory distress syndrome, sepsis, and even hematologic malignancies, can occur [12, 13].

Ehrlichiosis can be diagnosed through direct microscopic examination, serology, cultivation, and molecular techniques [14]. Smear-based diagnosis has low sensitivity, and serodiagnosis cannot differentiate between new and old infections, leading to false-negative results during the acute phase of infection [15, 16]. Currently, the only available alternative is polymerase chain reaction (PCR) using whole blood samples, the most sensitive method for diagnosing acute ehrlichiosis [17, 18].

*B. burgdorferi*, another spirochete-borne pathogen, causes Lyme disease, a zoonotic disease transmitted by hard ticks as intermediate host vectors [19]. *B. burgdorferi* is transmitted horizontally between ticks and reservoir hosts (wild animals and small mammals). Humans may become infected incidentally, and the clinical signs can vary depending on the bacterial strain. However, cold symptoms and expanding erythema are common in most human patients [20, 21]. Only approximately 5%–10% of *B. burgdorferi*-infected dogs show symptoms, making dogs potential reservoirs for the bacteria and contributing to the underestimation of Lyme disease prevalence, increasing the risk of disease dissemination [17, 22].

*C. burnetii*, another potential tick-borne bacterial pathogen, causes Q fever in humans, while animal infection is known as coxiellosis. It is a widely distributed zoonotic disease with significant negative impacts on animal welfare, human health, and economies [23, 24]. *C. burnetii* is a Gram-negative obligate intracellular rickettsial bacterium that predominantly replicates in host macrophages [25]. Although *C. burnetii* primarily infects its hosts through the inhalation of contaminated dust and aerosols, ticks are known to transmit the bacteria among animal hosts, serving as a reservoir for *C. burnetii*. A wide range of animal reservoirs act as sentinels for *C. burnetii*. Although the disease often goes unnoticed in animal hosts, it can cause serious clinical outcomes such as livestock abortion. Aborted materials are a significant source of human infection and environmental contamination, with one gram of placenta potentially containing 10^9^ bacteria. As a result, greater attention must be paid to livestock animals, such as cattle, sheep, and goats, for immunological investigations [26]. In addition, manure from domestic, wild animals, and pets, as well as ticks, is a common source of *C. burnetii* infection through various means [26].

Although most individuals infected with Q fever show mild symptoms, many develop severe symptoms, and the infection can be fatal in immunocompromised humans [26]. *C. burnetii* can be isolated in cell culture media, but growing it in a laboratory poses hazards, so its manipulation should be restricted to biosafety level 3 laboratories [27]. Alternatively, the organism can be identified through serodiagnosis, immunological testing of biopsy specimens, and PCR [28].

Since the aforementioned tick-borne zoonoses are of significant veterinary and medical importance, and companion dogs can be potential reservoirs or sentinel hosts for these pathogens, it is crucial to focus research on these pathogens in dogs and associated ticks in Egypt. This is particularly necessary given the extreme climatic changes the country is facing and the advent of sensitive and rapid molecular diagnostic techniques since the 1990s, which have made diagnosing tick-borne diseases more accurate and comparatively rapid.

The number of companion dogs is increasing in Egypt. A rise in tick infestation has been observed in dogs due to drastic climatic changes in the area. Accordingly, this study aimed estimate the prevalence of *B. burgdorferi, E. canis*, and *C. burnetii* in dogs and associated ticks in Cairo and Giza, Egypt. Conventional PCR assays, which are the most reliable diagnostic tools for One Health-related threat, were employed in the survey.

## Materials and Methods

### Ethical approval

The study protocol was reviewed and approved by the local guidance of the Research Ethics Committee of the Faculty of Veterinary Medicine, Cairo University (VET. CU.IACUC; approval no. Vet CU 131020241010)

### Study period and location

**Figure-1:**
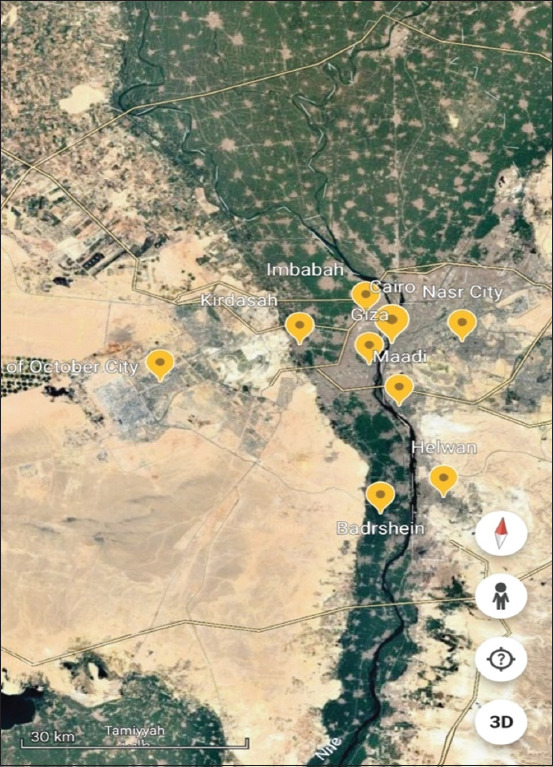
A Google map illustrating the sampling locations [Source: Google Earth].

Samples were collected from June 2023 to May 2024. The samples included blood from household and kenneled dogs as well as whole ticks from infested dogs at nine pet clinics in Cairo and Giza governorates, near the Faculty of Veterinary Medicine, Cairo University, as shown in Figure-1.

### Sample size

The sample size was determined using the formula described by Thrustfield [29], which is frequently employed in veterinary epidemiology and other disciplines. n = Z^2^*P*(1−P)/d^2^.

where (n) is the required sample size, (Z) is the Z-value (the number of standard deviations from the mean corresponding to the desired confidence level, e.g., 1.96 for 95% confidence), (P) is the estimated prevalence or proportion of the attribute being measured, (d) is the desired precision or margin of error.

### Collection of ticks

Ticks were collected from each dog in pools of approximately five ticks. Medium-sized steel forceps with blunt points were used to collect live and undamaged ticks for morphological analysis. The forceps, with serrated inner surfaces, were laid against the dog’s skin, and the ticks were pulled outward. Tick pools were placed in well-ventilated tubes with moistened paper, and cold tissue was sent to the laboratory with minimal delay [19].

### Data collection

A questionnaire was distributed to owners and veterinarians to obtain information about the dog’s size (small, medium, and large), type of coat (short hair or long hair), age (<3 years, ≥3–8 years, ≥8 years), gender, cohabitation with other dogs (pack size), type of housing (indoor or outdoor), contact with other pet or farm animals (dogs, cats, horses, and ruminants, history of tick infestation, and general ectoparasite control practices).

### Blood sampling

Whole blood was drawn from the cephalic vein of each tick-infested dog (110 samples) into ethylenediaminetetraacetic acid (EDTA)-coated tubes (Greiner Bio-One International GmbH, Austria). The blood samples were transported to the laboratory under cold conditions (4°C) for further processing. Small blood aliquots were stored at −20°C in 2 mL Eppendorf tubes (ThermpFisher, UK) for DNA extraction.

### Tick species identification

Adult ticks collected from dogs were examined under a stereomicroscope (Olympus, Japan). The identification was conducted as described by Walker [30]. Ticks from each pool were stored separately in sterile vials at −20°C for further analyses.

### DNA extraction

DNA was extracted from collected blood and whole tick samples using QIAamp DNA mini kits for blood and tissue samples (Qiagen, N.V., Hilden, Germany). The manufacturer’s instructions were fo- llowed, and the extracted DNA was quantified and frozen for subsequent PCR amplification assays.

### PCR detection of *B. burgdorferi, E. canis*, and *C. burnetii* in DNA extracted from whole ticks and dog blood samples

DNA samples extracted from blood and infes- ting whole ticks were screened for *B. burgdorferi* and *E. canis* using conventional PCR methods, whereas a nested PCR assay was employed for *C. burnetii* [30]. Primers were purchased from Metabion (Germany). The primer sequences and thermocycling conditions are listed in Table-1 [3, 31, 32]. The final volume of each PCR reaction was 50 μL, containing 5 μL DNA, 25 μL master mix (EmeraldAmp GT PCR, Takara, Japan), 1 μL of 10 μM of each primer, and 18 μL nuclease-free water (Qiagen, Germany). PCR amplification was performed using a thermal cycler (Techne^®^ Prime, UK). The amplification products were visua- lized through agarose gel electrophoresis in 1.5% agarose in 1× Tris-acetate-EDTA (BDH Limited Poole, England) buffer containing 0.5 μg/mL ethidium bromide, followed by gel examination under ultra-violet transillumination (Vilber Lourmat, France).

**Table-1:** Primers and thermocycling conditions for the polymerase chain reaction detection of *Borrelia burgdorferi*, *Ehrlichia canis*, and *Coxiella burnetii*.

Gene	Agent	Oligonucleotide sequences (5′-3′)	Temperature cycles	bp	References
Bb	*Borrelia burgdorferi*	BbF: GGG ATG TAG CAA TAC ATTC BbR: ATA–TAG TTT, CCA–ACA–TAG G	Initial denaturation for 1 min at 94°C 35 amplification cycles, 3 steps each: 1 min at 95°C, 1 min at 50°C, and 1.5 min at 72°C Final extension for 7 min at 72°C	577	[32]
PER	*Ehrlichia canis*	PER1:TTTATCGCTATTAGATGAGCCTATG PER2: CTCTACACTAGGAATTCCGCTAT	Initial denaturation for 5 min at 95°C 40 cycles at 95°C for 30 S; 55°C for 30 s and 72°C for 30 s Final extension at 72°C for 5 min	451	[3]
htpAB	*Coxiella burnetii*	IS111 F1: TACTGGGTGTTGATATTGC IS111 R1: CCGTTTCATCCGCGGTG,	Initial denaturation at 95°C for 3 min 40 cycles: 95°C for 30 s, and 52°C for 30 s at 72°C for 1 min Final extension at 72°C for 4 min	485	[31]
IS1111		IS111 F2: GTAAAGTGATCTACACGA IS 111 R2: TTAACAGCGCTTGAACGT	Initial denaturation at 95°C for 3 min Thirty cycles: 95°C for 30 s and 52°C For 30 s; and 72°C for 30 s) Final extension at 72°C for 4 min	260	

### Statistical analysis

Data were analyzed using descriptive statistics (frequencies) and the Chi-square (χ^2^) test for independence to investigate the relationship between tick infestation rates and sampling type. p < 0.05 was considered statistically significant.

## Results

### Identification of ticks

**Figure-2:**
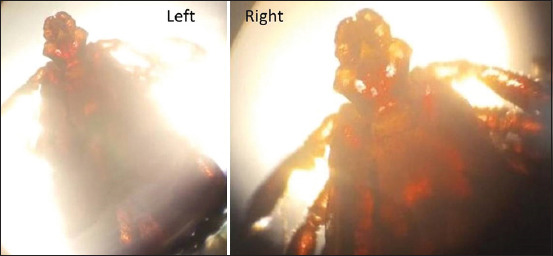
The dog-brown tick (*Rhipicephalus sanguineus*) collected from household and kenneled dogs. Photos showing the whole tick (left) and mouth with the front part (right) magnified using a stereomicroscope (10× magnification).

Based on macroscopic and microscopic morphological criteria, the only species identified from all dogs (110 pools) was *R. sanguineus* (Figure-2).

### PCR-based of *B. burgdorferi, E. canis*, and *C. burnetii* in *R. sanguineus* and dog populations

**Figure-3:**
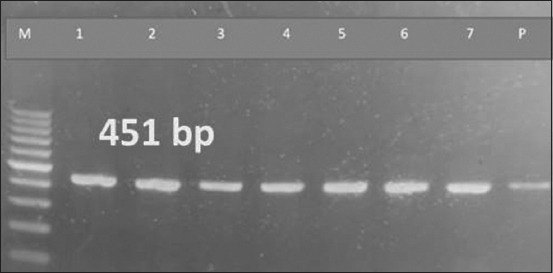
Polymerase chain reaction amplification results for specific detection of *E. canis* in dog blood samples and tick pools DNA based on the PER2 of *16S rRNA* gene primer. M: 100-bp DNA size marker; 1 and 2 lanes: Tick DNA; lanes 3–7: Dog blood DNA; and P: Positive control DNA. Lanes 1–7 show the positive 451-bp amplicons specific for *E. canis. E. canis=Ehrlichia canis*.

**Figure-4:**
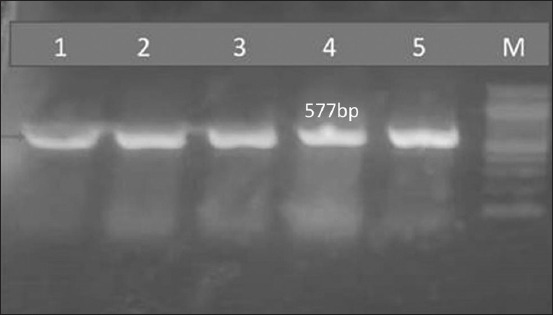
PCR analysis of the *B. burgdorferi* B*p* gene in DNA extracted from dog blood and brown tick pools. Lanes 1–4: Positive 577-bp amplicons specific for *B. burgdorferi* from household dogs and attached ticks; lane 5: positive control DNA and M: DNA size marker (100 bp). *B. burgdorferi=Borrelia burgdorferi*. PCR=Polymerase chain reaction.

Of 110 dog blood samples (40 households and 70 kenneled), *B. burgdorferi* DNA was detected in three dogs (one household and two kenneled). *E. canis* DNA was detected in six dogs (two household and four kenneled), while *C. burnetii* was detected in one kenneled dog. The differences in the prevalence of the three types of infection in dogs were not significant (χ^2^ = 0.012; p > 0.05, χ^2^ = 0.025; p > 0.05, respectively). Concerning the 110 associated tick pools, *B. burgdorferi* was detected in four pools (one from household and three from kenneled dogs), *E. canis* was detected in 12 pools (four from household and eight from kenneled) while *C. burnetii* was detected in three pools from kenneled dogs, with no significant difference noted (p > 0.05). The overall prevalence of the three agents in dog and tick samples were 3.18%, 8.18%, and 1.81%, respectively, with significant diff- erences (p < 0.05). Positive PCR reactions of dogs and their associated pools were encountered in four cases (Table-2 and Figures-2–4) of *B. burgdorferi* (1), *E. canis* (2), and *C. burnetii* (1).

**Table-2:** Prevalence of *B. burgdorferi, E. canis, and C. burnet*ii in *Rhipicephalus sanguineus* tick pools and dog populations.

Sample type	Rearing type and no. of samples	No. of samples and percentages of positive samples		

		*B. burgdorferi*	*E. canis*	*C. burnetti*
Dogs (110)	Household (40)	1 (2.5)	2 (5)	0 (0)
	Kenneled (70)	2 (2.85)	4 (5.7)	1 (1.42)
χ^2^		0.012	0.025	ND
p-value		0.911	0.8739	ND
Tick pools (110)	Household (40)	1 (2.5)	4 (10)	0 (0)
	Kenneled (70)	3 (4.28)	8 (11.42)	3 (4.28)
χ^2^		0.231	0.0534	ND
p-value		0.630	0.817	ND
Total	220	7 (3.18)	18 (8.18)	4 (1.81)
p-value		0.0064*		
Positive dogs and attached ticks	4	1	2	1

χ^2^=Chi-square test;

* =Significant at p *<* 0.05; ND=Not detected, *B. burgdorferi=Borrelia burgdorferi, E. canis=Ehrlichia canis, C. burnetti=Coxiella burnetii*

### Prevalence of co-infection by dogs with *B. burgdorferi, E. canis*, and *C. burnetii*

As depicted in Table-3, mixed infections with more than one of the three targeted pathogens in the same dog or the same tick pool were detected. Out of the 110 dog blood samples and 110 tick pools, *B. burgdorferi* and *E. canis* were simultaneously detected in two dogs and two tick pools, and *C. burnetii* was detected in one dog and none of the tick pools, whereas the three agents were all detected together in one dog and none of the tick pools (χ^2^ = 0.685; p > 0.05). Mixed infection with *C. burnetii* and *B. burgdorferi* was detected in one dog and one tick pool. The overall prevalence of mixed infection was 3.63% (8/220: five dogs and three tick pools) (χ^2^ = 0.204; p > 0.05).

**Table-3:** Prevalence of coinfection of dogs and associated ticks with *B. burgdorferi, E. canis*, and *C. burnetii*

Type and no. of samples	No. of positive *B. burgdorferi strains*	No. of positive *E. canis*	No. of positive *C. burnetii*	*E. canis* coinfected with the genus			*C. burnetii* co-infected with the genus *B. burgdorferi*	Total no. of samples with coinfection

				*B. burgdorferi*	*C. burnetii*	*B. burgdorferi +* *C. burnetii*		
Blood (110)	3 (3.3%)	6 (6.6%)	1 (1.1%)	2	1	1	1	2
Ticks (110)	4 (4.4%)	12 (13.2%)	3 (3.3%)	2	0	0	1	3
χ^2^	0.147	2.178	1.018	0.685			0	0.204
*p*-value	0.700	0.139	0.312	0.407			1	0.650

χ^2^=Chi-square test, *Significant at p *<* 0.05. *B. burgdorferi=Borrelia burgdorferi, E. canis=Ehrlichia canis, C. burnetti=Coxiella burnetii*

## Discussion

Ticks are obligate hematophagous ectoparasites that pose a significant risk to both animals and public health by potentially transmitting serious pathogens to infested hosts, which are known as TBPs [33].

Dogs have long been human companions, significantly affecting human life and drawing attention to the diseases that affect them. One of the most concerning threats to dogs is tick-borne diseases, which present a fundamental diagnostic challenge for veterinarians due to their non-specific clinical signs. In addition, coinfections with multiple pathogens intensify the challenge. At present, molecular methods are helpful for accurate diagnosis and describing the prevalence and epidemiology of tick-borne diseases in dogs. However, information on this issue is still lacking in many countries worldwide [34]. Egypt is a country where the prevalence and distribution of several critical TBPs in dogs require further investigation.

In this study, we screened blood samples from 110 tick-infested dogs (both household and kenneled) in Cairo and Giza governorates, Egypt, along with their associated ticks, using PCR to detect three different zoonotic bacterial pathogens: *B. burgdorferi, E. canis*, and *C. burnetii*. More than one tick was collected from each infested dog to create a pool. Morphological examination of the tick pools using a stereomicroscope revealed that all were of the species *R. sanguineus*, the brown dog tick (Figure-2). This indicates that the species is widely distributed and is nearly the only species infesting dogs in the study region. It has been reported that *R. sanguineus* is the most widespread tick, infecting dogs in both urban and rural areas [35].

Based on the PCR analysis of dog blood samples and tick pools, the prevalence rates of *B. burgdorferi* in household and kenneled dogs were 2.5% and 2.81%, respectively (Table-2 and Figure-3). In contrast, studies by Elhelw *et al*. [19] and Senbill *et al*. [36] reported prevalence rates of dog borreliosis in Egypt at 23% and 1.67%, respectively. These differences could be attributed to geographical location, the season of sample collection, the physiological state of the dogs, and the level of care provided by owners or shelter workers.

Regarding tick pools, *B. burgdorferi* was detected in 2.5% and 4.28% of ticks collected from household and kenneled dogs, respectively (Table-2). The increased prevalence of *B. burgdorferi* among ticks in dogs may be attributed to the fact that these dogs are usually gathered from different places, potentially harboring infected animals with tick infestations. A higher prevalence of *B. burgdorferi* was recorded in dog ticks in previous studies by Elhelw *et al*. [19] and Senbill *et al*. [36]. However, those studies involved fewer ticks than the number investigated in this study. The lower prevalence observed in our study could be attributed to the larger sample size, increased awareness of this problem among pet owners and veterinarians, and the care provided to pet dogs. Our results indicated that *B. burgdorferi* is prevalent in dogs and associated ticks in the study area; both pose a potential source of zoonosis dissemination to humans, dogs, and other animals [37, 38].

The second tick-borne bacterial pathogen targeted in this study was *E. canis*, which was detected by PCR in 5% and 5.7% of blood samples collected from households and kenneled dogs, respectively (Table-2 and Figure-4). The prevalences of *E. canis* in collected ticks from household and kenneled dogs were even higher at 10% and 11.4%, respectively. Although these prevalences are comparatively high, Juasook *et al*. [39] reported much higher prevalence (64% and 82% in dogs and ticks, respectively) as detected by PCR. However, our results are similar to Juasook *et al*. [39] in two aspects: first, the brown dog tick (*R. sanguineus*) was the only species found in both studies in Egypt and Thailand, and second, PCR was the screening tool in both studies, with *E. canis* prevalence being higher in ticks than in dogs in both investigations.

Serologic evidence from previous studies indicates that *E. canis* is widespread among dogs worldwide. The seroprevalence ranged from 30% to 80% in African countries [40–44], whereas in some Asian countries, the prevalence ranged from 0.2% to 30% [45, 46]. Even in Europe, the prevalence ranged from 2% to 50%, and in the USA, *Ehrlichia* antibodies were detected in 1.3% of dogs in the Southeast, whereas other regions showed lower rates, estimated between 0.3% and 0.6% [47].

In a similar study in Egypt, the prevalence of *E. canis* was 2.9% and 1.4% in household and kenneled dogs, respectively [3]. Variable prevalence rates could be attributed to screening tools, geographical distribution, and surveyed dog species. The higher infection level observed in our study is comparable with that by Asmaa *et al*. [3] may be related to climatic changes, with increased temperatures leading to an increase in the tick population in Egypt.

The brown dog tick (*R. sanguineus*) acts as the primary vector of *E. canis*, transferring the pathogen between hosts during blood meal feeding. Both domestic and wild dogs serve as reservoir hosts for this pathogen and are the primary sources of brown dog ticks. Brown dog ticks become carriers of the pathogen when feeding on an infected dog. *E. canis* is stored in the midgut and salivary glands of infected ticks and is transferred through saliva to the host during blood meals [47]. Our study found prevalences of *R. sanguineus* and *E. canis* at 10% and 11.4% in household and kenneled dogs, respectively (Table-2 and Figure-3). A higher result (82%) was recorded by Juasook *et al*. [39], while lower results were reported by Asmaa *et al*. [3], who found 1.94% and 1.4% in household and kenneled dog ticks, respectively.

**Figure-5:**
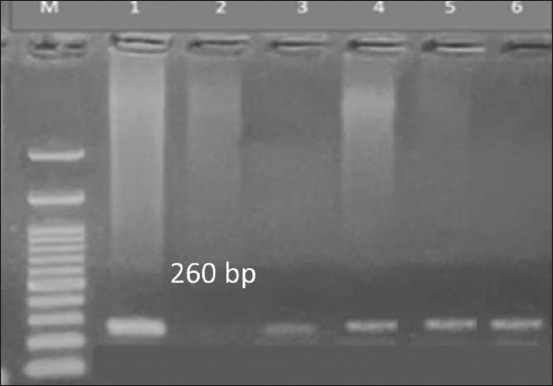
Nested PCR detection of the *Coxiella burnetii* htpAB-IS1111 gene in DNA extracted from dog blood and attached brown tick pools. M: DNA size marker (100 bp); lanes 3–6: DNA from dog blood samples and tick pools showing positive 260-bp specific amplicons; lane 1: Positive control DNA and lane 2: Negative control (master mix, primers, and nuclease-free water). PCR=Polymerase chain reaction.

Q fever is a severe tick-borne disease caused by *C. burnetii*, an intracellular, small Gram-negative coccobacillus that is highly resistant to high temperatures [48]. Q fever is often neglected as a zoonotic disease in many developing countries, and there is no published data regarding the prevalence of *C. burnetii* in companion animals such as dogs and cats in Egypt. In this study, we estimated the prevalence of *C. burnetii* in blood samples collected from tick-infested dogs using a nested PCR assay. We detected *C. burnetii* DNA in 1.4% of kenneled dogs and 4.2% of their attached ticks, whereas household dogs and their attached ticks were negative (Table-2 and Figure-5). In another study, *C. burnetii* was detected in 11% of blood samples from dogs using nested PCR [49], whereas a lower prevalence of 0.55% was reported by Norris *et al*. [50].

The seroprevalence of *C. burnetii* infection in dogs ranges from 0% to 35% based on enzyme-linked immunosorbent assay or immunofluorescence assay tests conducted in New SouthWales, Australia [51]. Seroprevalence of *C. burneti* was reported at 30% in dogs in Brazil [52], 20.3% in stray dogs in Southern Hungary [53], and 5.5% in feral canines in Iraq [54]. The variations in the prevalence of *C. burnetii* across different reports may be attributed to host factors, geographical and environmental conditions, and detection methods.

Dogs can acquire *C. burnetii* infection through inhalation, consumption of infected milk, placentas, or carcasses, or following tick bites. Infected animals can transmit Q fever to humans during or after parturition [51]. This was demonstrated in cases of human *C. burnetii* pneumonia following contact with an infected parturient bitch [55].

Outdoor housing of dogs, which increases their contact with farm animals, wildlife, and ticks, as well as feeding them a raw diet, may contribute to the higher prevalence of Q fever. Outdoor housing provides greater opportunities for dogs to contract infection from other animals. In addition, feral dogs are more susceptible to pathogens due to poor diet, improper environment, and deficient immunity [49].

In this study, we detected coinfections with two TBPs in three kenneled dogs: two were infected with *E. canis* and *B. burgdorferi*, and one was infected with *C. burnetii* and *B. burgdorferi* (Table-3). This finding is not surprising, as dogs already infected with one pathogen are more likely to be susceptible to infection with another. Mixed infections were reported in two pooled tick samples: One contained *E. canis* and *B. burgdorferi*, and the other contained all three bacterial pathogens (*E. canis, C. burnetii*, and *B. burgdorferi*). Moreover, the four dogs had the same bacterial pathogens as those in their attached ticks (Table-3).

Little *et al*. [17], Smith *et al*. [22], and Abdel-Moein *et al*. [26] reported prevalences of 1.4%, 0.5%, and 6.7% for ehrlichiosis, borreliosis, and coxiellosis, respectively in dogs. Nevertheless, as analyzed by the Chi-square test, the variables detected in our study were insignificant within samples of the same category. In other words, there were no significant differences in the prevalence of bacterial infection between household and kenneled dogs or between the associated ticks. However, significant differences were observed for borreliosis among dogs and their associated ticks (Tables-2 and 3).

Coinfections can complicate diagnostics and may modulate disease severity through synergistic effects. Summarizing the different types of coinfections may ease the diagnostic challenges raised by exposure to multiple pathogens and pave the way for effective treatment [56]. PCR can simultaneously detect natural co-infections with tick-borne bacteria in dogs, making it a valuable tool for epidemiological mapping of various TBPs. It can help identify causative agents in the early stages of infection and evaluate treatment responses [57].

Human bites from the brown dog tick (*R. sanguineus*) are relatively rare. However, certain factors, such as dog ownership, daily occupational handling of dogs, and high environmental tick populations, increase the risk of brown dog tick parasitism in humans. Reports of human beings bitten by this tick species are increasing, especially in countries with warm climates, including Egypt. Therefore, people in these regions are more likely to contract the pathogenic agents that this species carries [3].

Unfortunately, we did not perform a sequencing analysis to identify *Coxiella* at the species level to confirm it as *C. burnetti*. This was due to a lack of sufficient funding for the study. This will be considered in a future investigation.

## Conclusion

The PCR assays employed in this study effectively screen dogs and their infesting ticks for the three targeted tick-borne diseases. The dog-brown tick (*R. sanguineus*) was the only species identified in dogs in the study area. Household and kenneled dogs and their attached ticks serve as reservoir hosts for *B. burgdorferi, C. burnetii*, and *E. canis*, which pose significant zoonotic threats to humans. *E. canis* showed the highest prevalence in both dog and tick samples followed by *B. burgdorferi* while *C. burnetti* showed the lowest prevalence. In addition, other zoonotic TBPs, which were not examined in this study, could intensify the situation. There is an urgent need for authorities and private organizations in Egypt to regulate dog ownership and implement control and prevention programs through continuous education and workshops.

## Authors’ Contributions

MEH and ZSA: Designed the study. NH: Methodology and investigation. TMA and HM: Sample collection, drafted and revised the manuscript and statistical analysis. All authors have read and approved the final manuscript.
